# Glypican-1 in human glioblastoma: implications in tumorigenesis and chemotherapy

**DOI:** 10.18632/oncotarget.27492

**Published:** 2020-03-03

**Authors:** Eduardo Listik, Leny Toma

**Affiliations:** ^1^Department of Biochemistry, Universidade Federal de São Paulo, São Paulo, SP, Brazil

**Keywords:** glioblastoma, glypican-1, tumorigenesis, chemotherapy resistance, temozolomide

## Abstract

Glioblastoma is one of the most common malignant brain tumors, with which patients have a mean survival of 24 months. Glypican-1 has been previously shown to be overexpressed in human glioblastoma and to be negatively correlated with patient’s survival. This study aimed to investigate how glypican-1 influences the tumoral profile of human glioblastoma using *in vitro* cell line models. By downregulating the expression of glypican-1 in U-251 MG cells, we observed that the cellular growth and proliferation were highly reduced, in which cells were significantly shifted towards G0 as opposed to G1 phases. Cellular migration was severely affected, and glypican-1 majorly impacted the affinity towards laminin-binding of glioblastoma U-251 MG cells. This proteoglycan was highly prevalent in glioblastoma cells, being primarily localized in the cellular membrane and extracellular vesicles, occasionally with glypican-3. Glypican-1 could also be found in cell-cell junctions with syndecan-4 but was not identified in lipid rafts in this study. Glypican-1-silenced cells were much more susceptible to temozolomide than in U-251 MG itself. Therefore, we present evidence not only to support facts that glypican-1 is an elementary macromolecule in glioblastoma tumoral microenvironment but also to introduce this proteoglycan as a promising therapeutic target for this lethal tumor.

## INTRODUCTION

The most frequent central nervous system (CNS) malignant tumors are the gliomas, which arise from glial precursor cells, and can be divided into oligodendrogliomas, oligoastrocytomas, ependymomas, astrocytomas, and some minor classifications [1]. According to the World Health Organization’s (WHO) grading, glioblastoma (GBM) is a grade IV astrocytoma, which happens to have a highly-prevalent incidence rate. In the United States alone, 3.21 inhabitants per 100,000 presented the disease, as 14.7% of all brain tumors or even 56.6% of the gliomas are glioblastomas. GBM is incurable, and patients have a mean survival of 24 months while in treatment, rarely reaching up to 5 years [2]. The palliative treatment involves surgical resection of possible areas of the tumor followed by radiotherapy and chemotherapy with temozolomide (TMZ) [3].

The tumor environment possesses significant metabolic and structural differences from healthy tissue. Proteoglycans (PGs) are a class of macromolecules that show distinct patterns in cancers as opposed to physiological conditions [4]. PGs are structured by a core protein and linked chains of polysaccharides named glycosaminoglycans (GAGs) [5]. GAGs are made up by disaccharide repeats and specific glycosidic bonds. Heparan sulfate (HS), for instance, possesses mainly disaccharide repeats of glucuronic acid and N-acetylglucosamine. Other GAGs are chondroitin sulfate, dermatan sulfate, keratan sulfate, hyaluronic acid, and heparin [6].

Heparan sulfate proteoglycans (HSPGs) are known to participate in various aspects of cell signaling. The syndecans (SDCs), with four isoforms, and glypicans (GPCs), with six isoforms, are membrane-bound and generally located in lipid rafts; they can interact with morphogens such as ligands from the Wnt, Hh, and FGF families in a manner to facilitate interaction with their receptors [7]. These HSPGs are also essential elements of cell migration and adhesion, due to their interaction with integrins, elements from the extracellular matrix and chemokines [8].

Glypican-1 (GPC1) is, along with other GPCs, bound to the membrane through a glycosylphosphatidylinositol (GPI) anchor [9]. It is ubiquitous and influences cell-cell and cell-matrix adhesion, metastasis, invasion, and proliferation in various tumors, such as breast, pancreatic, and esophageal cancers [10–18]. Although GPC1 has received little focus in regard to glioblastoma, Saito & colleagues demonstrated how this molecule influences the lower survival of GBM patients [19]. Additionally, it has been previously shown that GPC1 may affect FGF-2 signaling in this tumor, contributing to its proliferative aspect and aggressiveness [20].

Our objective in this work is to elucidate further the role of GPC1 in this menacing tumor. To that end, we would deplete GBM cells of the molecule and investigate biological, biochemical, and pharmacological aspects of the modified cell lines to obtain further answers of how this HSPG could influence the tumorigenic process of GBM.

## RESULTS

### Selection of GBM cells and knock-down clones

GPC1 was highly expressed in the tested GBM cell lines, but there was no differential expression between them (Supplementary Figure 1A). By analyzing GPC1 core protein expression in T98G, U-251 MG, and U-373 MG cells, it was clear that all three cells have similar levels of the PG (Supplementary Figure 1B and 1C). We selected the U-251 MG cell line for the downstream experiments as these cells were much more proliferative and could reveal better discrepancies after knock-down of GPC1.

Lentiviral transduction effects were analyzed by RT-qPCR, and the generated polyclonal cell lines were investigated for either GPC1 or GAPDH gene expression (Supplementary Figure 2). The positive control (C+), GAPDH, was silenced in 98%, revealing high transduction efficiency. Only MOI 20 and 30 induced significant GPC1 silencing that differs from the negative control (C-). Nevertheless, the MOI 30 polyclonal cell line was selected for the monoclonal selection due to higher probability and number of integration events per cell.

**Figure 1:**
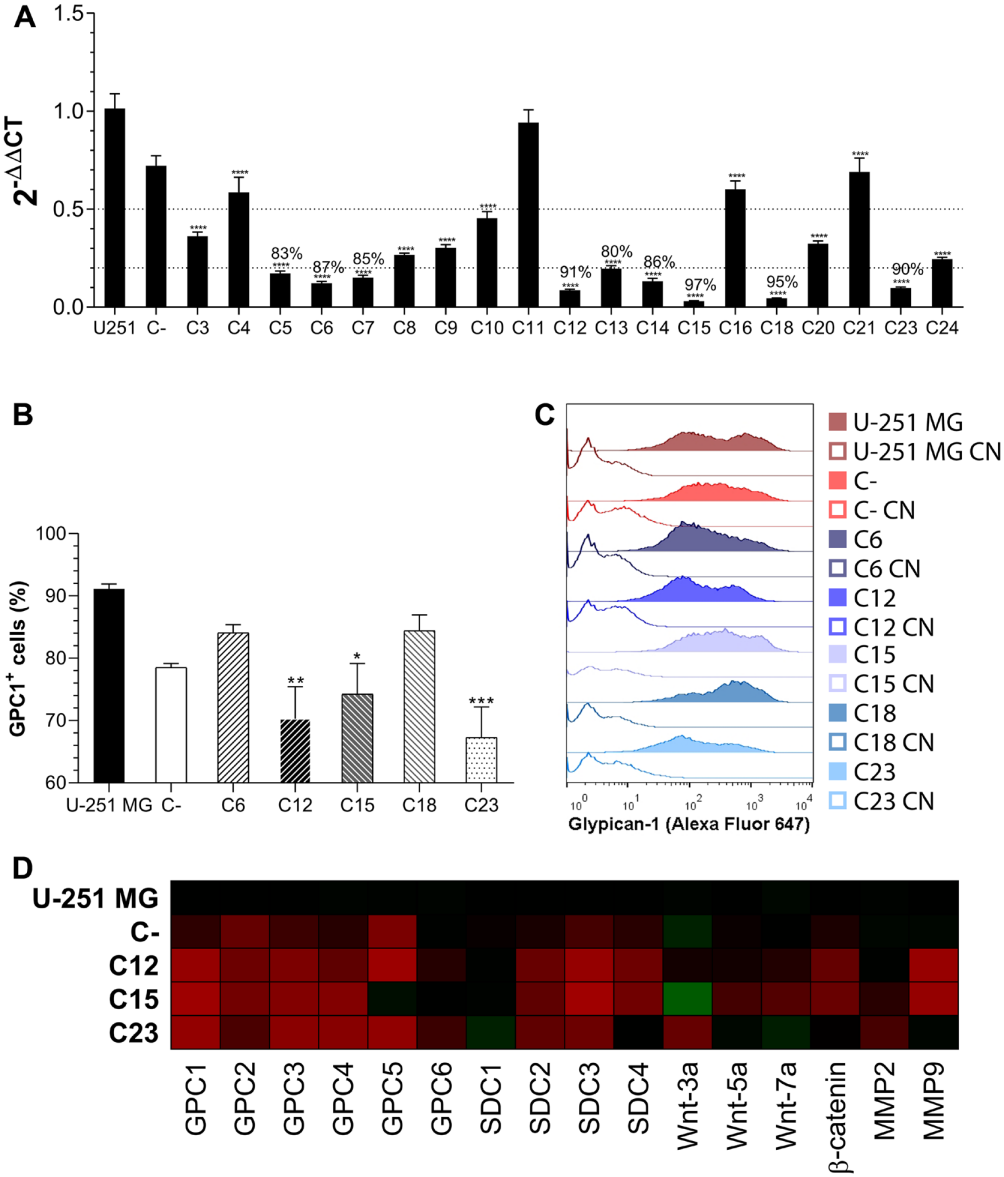
GPC1 knock-down clone selection. (**A**) All generated monoclonal cell lines had the GPC1 expression quantified by RT-qPCR. Dotted lines represent 50% and 80% of GPC1 silencing, respectively and, when more than 80% of gene silencing was achieved, the percentage reduction is indicated. (**B**) Flow cytometry assessment of GPC1 in control GBM cells and the five most GPC1-silenced clones. This representation shows the fraction of GPC1^+^ cells for each group. (**C**) Representative histograms of GPC1 fluorescence intensity distribution. Unstained samples are represented by unfilled curves (controls, CN). (**D**) Transcriptional profile of membrane-bound HSPG, selected Wnt ligands, and MMPs that were assessed by RT-qPCR. The heatmap of 2^-ΔΔCt^ was generated, in which significant comparisons are not indicated but are commented on in the text. Gradients of red indicate diminished expression, and of green heightened expression in relation to U-251 MG. All data are plotted as mean ± SEM. The one-way ANOVA with the Dunnett’s post-hoc test was performed, and statistically significant comparisons are marked as follows: ^*^ *p* < 0.05, ^**^ *p* < 0.01, ^***^ *p* 0.001 and ^****^ *p* < 0.0001 *vs.* U-251 MG. The sample size was *n* = 6 for RT-qPCR and *n* = 5 for flow cytometry.

Monoclonal cell lines were generated, and GPC1 expression was assessed by RT-qPCR (Figure 1A). The five most-silenced clones had between 87–97% of GPC1 silencing and were subjected to GPC1 flow cytometry profiling (Figure 1B, 1C). The clones C12, C15, and C23 did exhibit fewer GPC1^+^ cells as well as a broader range of cells expressing lower levels of GPC1. Therefore, these three clones were chosen for subsequent assays.

### GPC1 depletion alters gene expression of selected HSPGs and related molecules

After selecting silenced GPC1 clones (C12, C15, and C23), RT-qPCR analysis was performed to measure selected membrane-bound HSPGs’ expressions (all GPCs, from 2 to 6, and SDCs, from 1 to 4). Control cell lines were the original U-251 MG cells and the C- transduced polyclonal cell line, the negative control. Gene expression was first compared to β-actin (2^-ΔCt^) and then to U-251 expression levels (2^-ΔΔCt^; Figure 1D).

The GBM cells mainly express GPC1, -4 and -6, and all SDCs (Supplementary Figure 3A). There is considerable variation in several HSPGs’ expression after silencing of GPC1; however, only SDC2 and -3 significantly had an inhibited expression after GPC1 knock-down, and SDC4 did reveal substantial reduction effects, but not in all clones. GPC6 was the only HSPG that was not influenced at all by the procedure, and C23’s SDC1 expression was enhanced.

In an attempt to follow our group’s lead in establishing a role between GPCs and Wnt signaling, we also checked the expression of Wnt-3a, -5a and -7a ligands as well as β-catenin. Wnt-5a was the major expressed Wnt ligand (Supplementary Figure 3B), yet none of the ligands revealed any pattern associated with GPC1 expression change, although β-catenin, which is highly expressed, was significantly less present in C12 and C15.

As GBM is frequently associated with extracellular matrix remodeling, we checked the expression of metalloproteinases (MMPs) 2 and 9. Although MMP2 was the major MMP expressed (Supplementary Figure 3C), a significant reduction was verified in MMP9. It is also possible to state that MMP2 did experience an expression alteration from GPC1 knock-down, although no statistically significant changes were noted between specific samples.

### GPC1-silenced GBM cells reveal slower growth rates and reduced proliferation

After verifying an overall expression profile change mediated by GPC1, we proceeded to investigate how the proteoglycan would affect the tumor growth and its cells’ proliferation.

**Figure 2:**
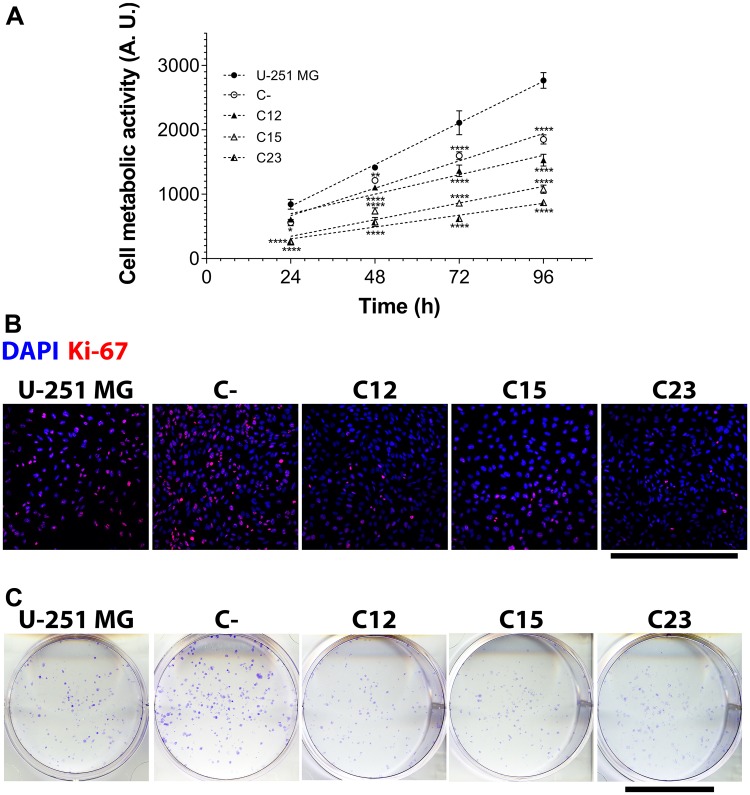
Cell metabolic activity, proliferation, and clonogenicity assays to assess GPC1 effects in GBM cells. The experiments were performed in U-251 MG, C- (both control cell lines) and C12, C15, and C23 GPC1 knocked-down cell lines. (**A**) The metabolic activity assay included reaction with MTT to obtain a growth curve by assessing cell metabolic activity at 24, 48, 72, and 96 h. Linear regression was done, and the obtained parameters are exhibited in Supplementary Table 1. Data are plotted as mean ± SEM, in which the sample size was *n* = 14. The two-way ANOVA with Dunnett’s post-hoc test was performed, and significant comparison are marked as follows: ^*^ *p* < 0.05; ^**^ *p* < 0.01; and ^****^ *p* < 0.0001 vs. U-251 MG. (**B**) Cells were immunolabeled with anti-Ki-67 antibody and additionally stained with DAPI for nuclear visualization to quantify proliferating cells (Ki-67^+^ cells). Images were obtained with a Leica TCS SP8 CARS confocal microscope. The scale bar refers to 500 µm. (**C**) To investigate whether the clonogenic potential was influenced by GPC1, 400 cells were plated in 6-well plates, incubated for eight mitotic cycles, and then stained with crystal violet. Only formations with more than 50 cells were considered colonies. The scale bar indicated 2 cm.

By constructing a growth curve of GPC1-silenced cells and control cells for up to 96 h (Figure 2A) and comparing them, it was clear that the knock-down reflected a reduction of 44.8–68.6% in the final metabolic activity. Using linear regressions, we did obtain the growth rate of each GBM cell line, and GPC1 downregulation could instigate a slowdown in cell growth of up to 71.5% (Supplementary Table 1).

The effects seen in cell metabolic activity instigated us to analyze cell proliferation, the clonogenic potentials, and cell cycles of these cell lines. For cell proliferation, we stained cells with Ki-67 and counted reactive nuclei, which indicates for proliferating cells (Figure 2B). It was evident that the knocked-down clones displayed a lower Ki-67^+^ fraction (a 43.7–48.0% reduction) and Ki-67^+^ cell density (a 40.4–54.8% reduction), as seen in Supplementary Figure 4A and 4B. Regarding clone formation, GPC1 also affected the ability of single cells to generate clones of a minimum of 50 cells (Figure 2C). C15 and C23 were critically affected by the absence of GPC1 and displayed modest numbers of formed colonies (Supplementary Figure 4C).

**Figure 3:**
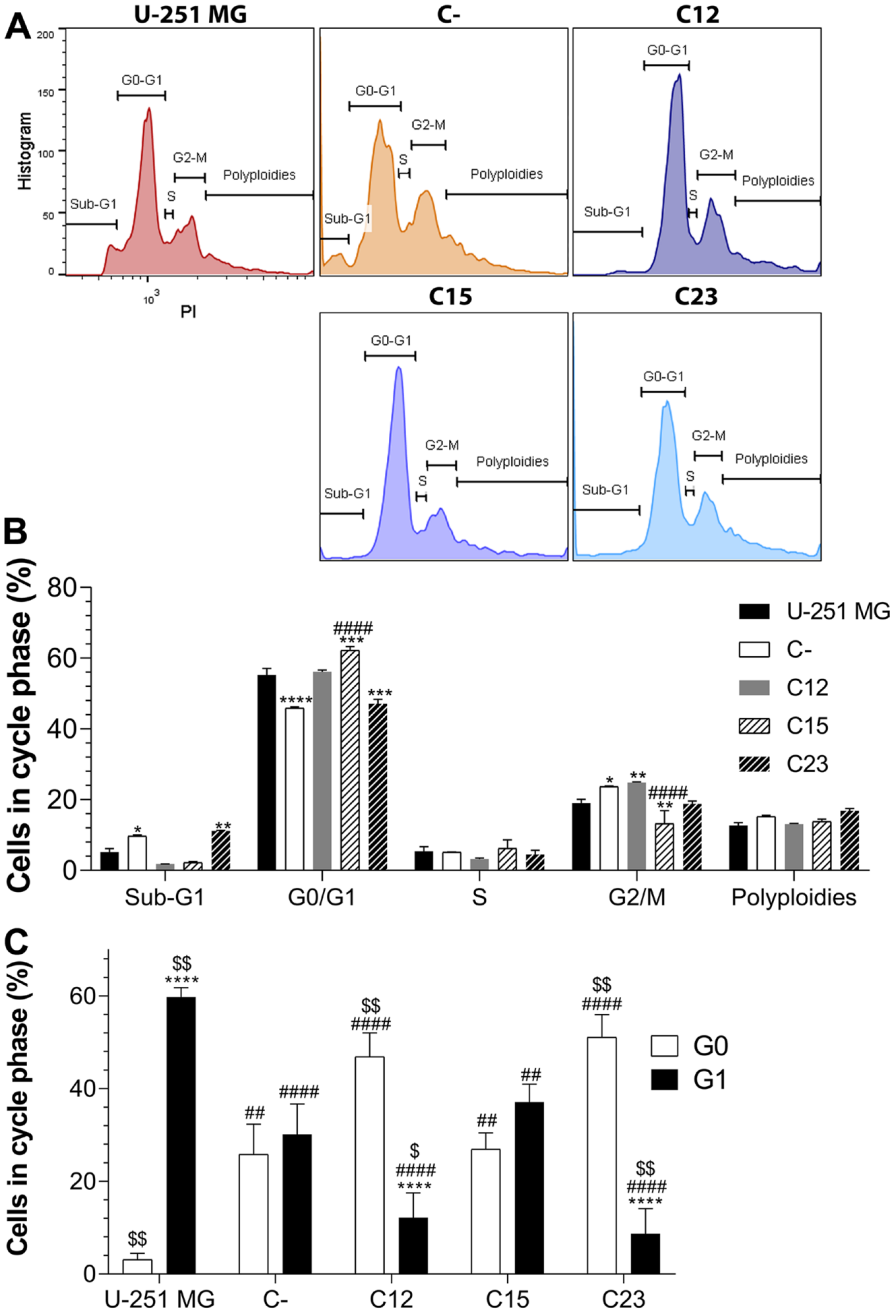
Cell cycle analysis to assess GPC1 influence on GBM. The experiments were performed in GBM control cells (U-251 MG, C-) and GPC1-silenced GBM cells (C12, C15, and C23). (**A**) Cells were stained with propidium iodide (PI) and divided between sub-G1, G0/G1, S, G2/M and polyploidies groups. Representative histograms are shown for each cell line. (**B**) Distribution of cells in each group and cell phases. All data are plotted as mean ± SEM, and flow cytometry was conducted in duplicate. The two-way ANOVA with Dunnett’s post-hoc test was performed, and statistically significant data are marked as follows: ^*^ *p* < 0.05, ^**^ *p* < 0.01, ^***^ *p* < 0.001, ^****^ *p* < 0.0001 *vs.* U-251 MG; and ^####^ *p* < 0.0001 *vs.* C-. (**C**) The fraction of cells in G0 and G1 phases when considering proliferation data. The sample size was *n* = 14. The two-way ANOVA with Bonferroni or Dunnett’s post-hoc test was done, and statistically significant comparisons are coded as follows: ^****^ *p* < 0.0001 *vs.* G0/G1; ^##^ *p* < 0.01, ^####^ *p* < 0.0001 *vs.* U-251 MG respective phase; and ^$^ *p* < 0.05, ^$$^ *p* < 0.01 *vs.* C- respective phase.

After verifying the profound effects on cell metabolic activity and proliferation, we investigated the cell cycle of the GBM cell lines (Figure 3). At first, no clear evidence in changes among the fractions for each phase was noted, aside from a reduced population of cells in G2/M in the C15 cell line (Figure 3A and 3B). Nonetheless, when considering the effects of proliferation, essential findings were encountered regarding the G0/G1 phases (Figure 3C). U-251 MG, in which almost 96% of the cells were found to be proliferating, displayed a panorama in which cells are mainly in G1, and infrequent in G0. This result was the opposite of other cell lines, especially C12 and C23, in which cells were mostly in G0. Thus, there seems to be a shift between the G0 and G1 phases induced by the absence of GPC1.

### GPC1 is infrequent in lipid rafts and has moderate association with other membrane-bound HSPGs in GBM

By affecting cell metabolic activity and proliferation, we opted to study the localization of GPC1 and association with selected HSPGs. GPC1 has been suggested to present itself in the lipid raft of skeletal muscle cell lines [21]; therefore, we attempted to identify this PG in these microdomains of GBM cells as well. Additionally, previous studies have revealed an intrinsic association between GPC1 and GPC3; or GPC1 and SDC4; thus, we sought to analyze these relationships in our experiments as well [22, 23].

**Figure 4:**
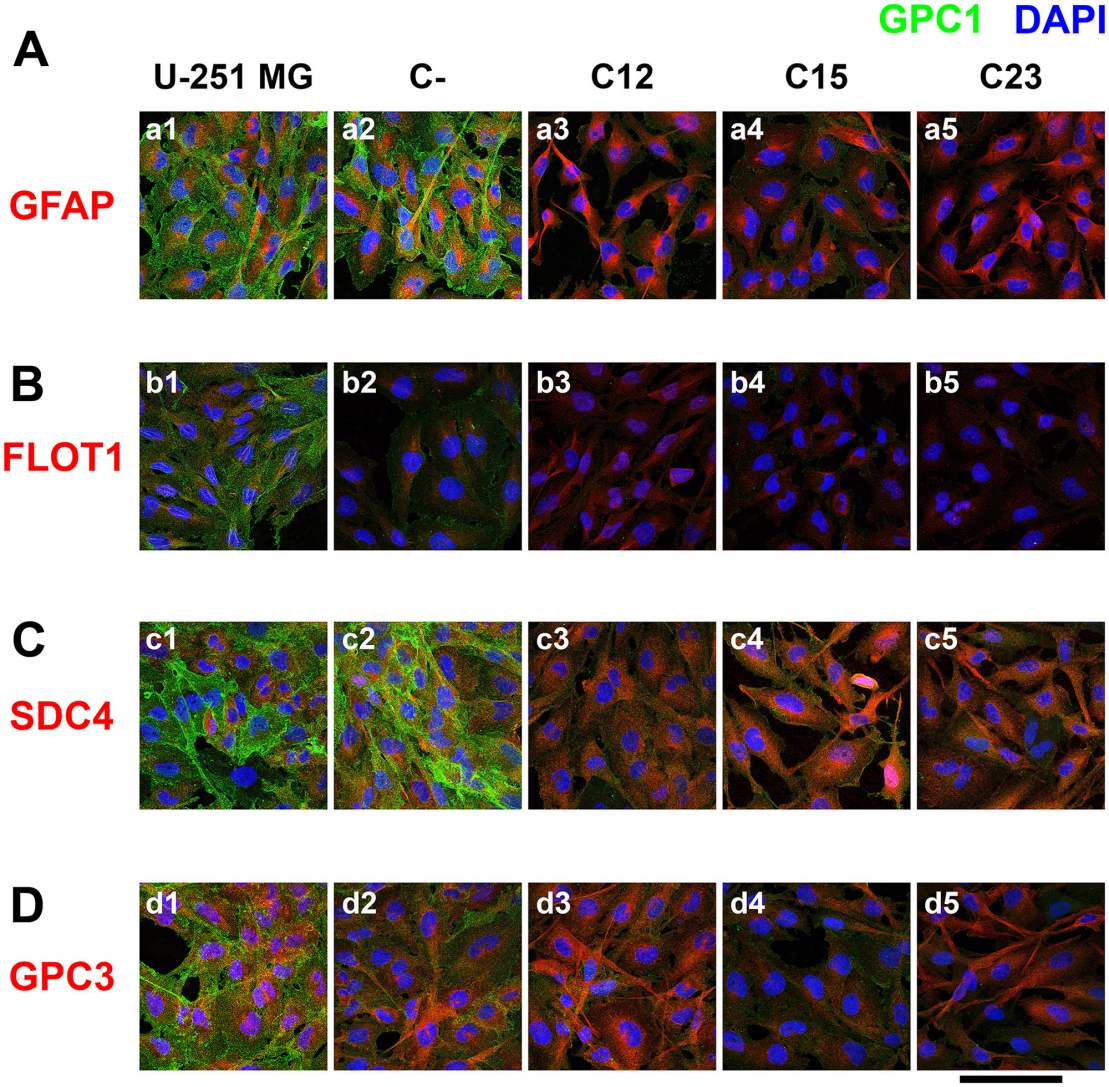
Confocal microscopy of immunolabeled GBM control and GPC1-silenced cells for a lipid raft marker or correlated HSPGs. U-251 MG (1), C- (2), as controls cells, and C12 (3), C15, (4) and C23 (5) GPC1-depleted cells were immunolabeled for GPC1, in green, and different other antigens, in red, such as (**A**) GFAP, (**B**) FLOT1, (**C**) SDC4, and (**D**) GPC3. The cells’ nuclei were stained with DAPI, shown in blue. The images were obtained with a Leica TCS SP8 CARS confocal microscope. The scale bar refers to 100 µm.

**Figure 5:**
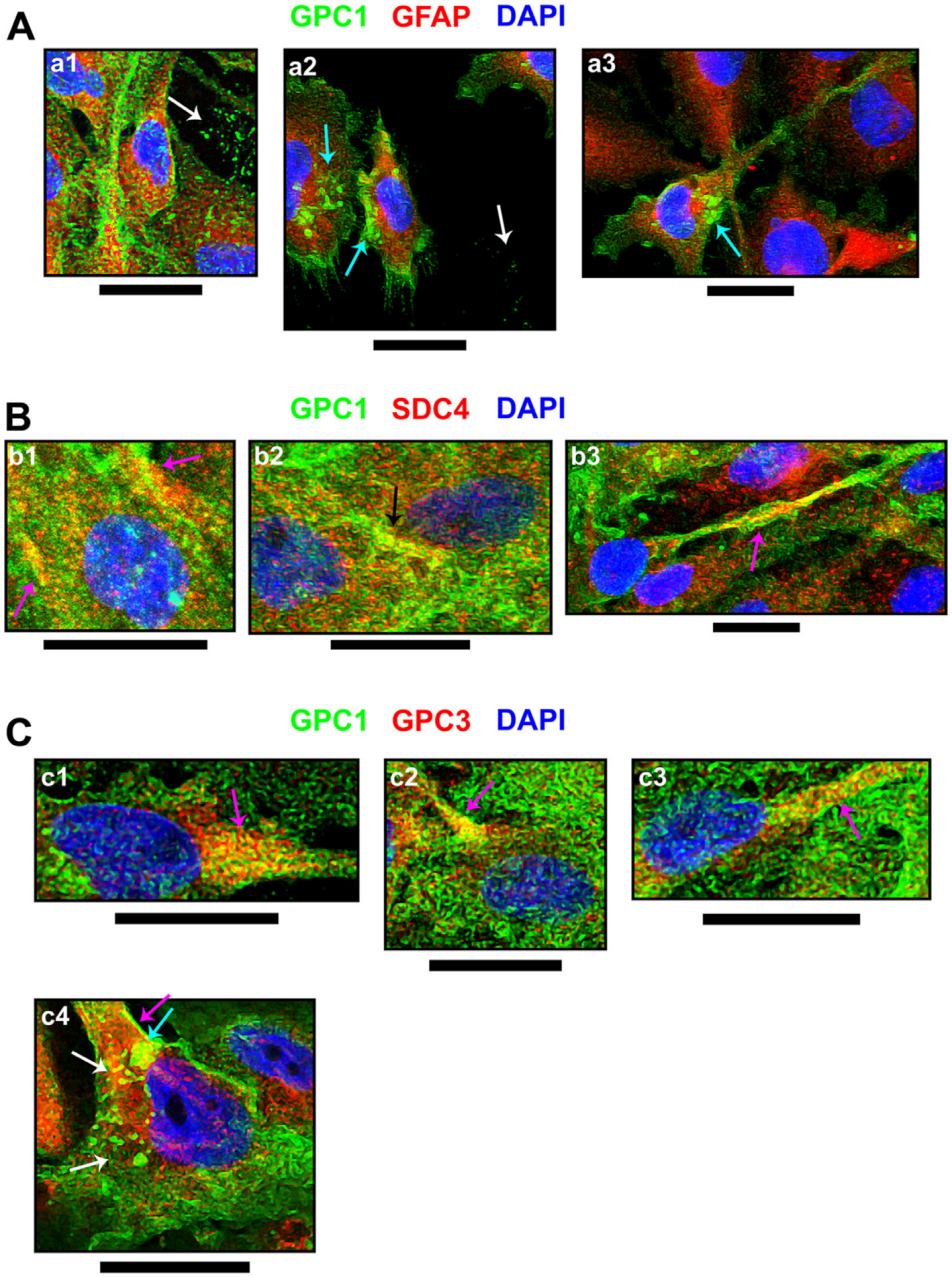
Cellular events indicating GPC1 and its association with SDC4 or GPC3. From immunofluorescence assays, through confocal microscopy, special events including (**A**) GPC1 and its co-localization with (**B**) SDC4 or (**C**) GPC3, both in red, were imaged. GPC1 is always represented in green, and DAPI staining for cells’ nuclei is in blue. Images were obtained from U-251 MG cells (b2, b3 and c1-c4) or C- (a1-a3 and b1) as there was evident GPC1 expression in these cell lines. Arrows have the following color code: detection in extracellular vesicles (white); extracellular vesicles’ interaction with the cellular membrane (cyan); co-localization in the cell membrane (magenta); co-localization in cell-cell junctions (black). The images were obtained with a Leica TCS SP8 CARS confocal microscope. All scale bars refer to 25 µm.

GPC1 was identified mainly in the cell membrane, and a significant reduction of 69.2–72.6% in knocked-down cell lines was observed (Figure 4A and Supplementary Figure 5A). Interestingly, GPC1 is highly present on extracellular vesicles (EVs) that were identified outside of the cellular body and in interaction with the cellular membrane (Figure 5A).

Our research on GPC1’s relation with lipid rafts was not convincing (Figure 4B). In U-251 MG cells, the Pearson correlation coefficient (PCC) was virtually zero (0.05 ± 0.05). Additionally, flotillin-1 (FLOT1), the lipid raft marker employed, displayed reduced expression in C15 and C23 (Supplementary Figure 5B). These results may indicate that, although GPC1 is minimally located in lipid rafts.

This effect was also observed with SDC4 and GPC3 (Supplementary Figure 5C and 5D). With SDC4, there was a homogenous protein expression of the SDC despite GPC1 downregulation (Figure 4C). SDC4 did colocalize much more with GPC1 than FLOT1, revealing a PCC of 0.16 ± 0.02 in U-251 MG (Supplementary Figure 5E). The colocalized patterns were found in the cell membrane, especially in cell-cell junctions (Figure 5B). The highest colocalization obtained was for GPC3 (0.20 ± 0.04 in U-251 MG). GPC3 appeared to be associated with GPC1 in the cell membrane and EVs (Figure 5C). Nevertheless, GPC3 was much less expressed than GPC1 (Figure 4D) and did show a pattern of reduced expression in GPC1-silenced clones as opposed to U-251 MG cells (Supplementary Figure 5D). However, as this reduction was not distinct from the levels of GPC3 obtained in the C-, we infer that GPC3 modulation may not be due to GPC1 itself.

The confocal microscopy results indicate how overly expressed GPC1 is in GBM cells. It is located in the cell membrane, is rarely found in lipid rafts, and can be colocalized along with SDC4 and GPC3. SDC4 and GPC1 are primarily found in cell-cell junctions, and GPC3 may be found altogether with GPC1 in EVs.

### Clones reveal migration hindrance and different interactions with extracellular matrix substrates

**Figure 6:**
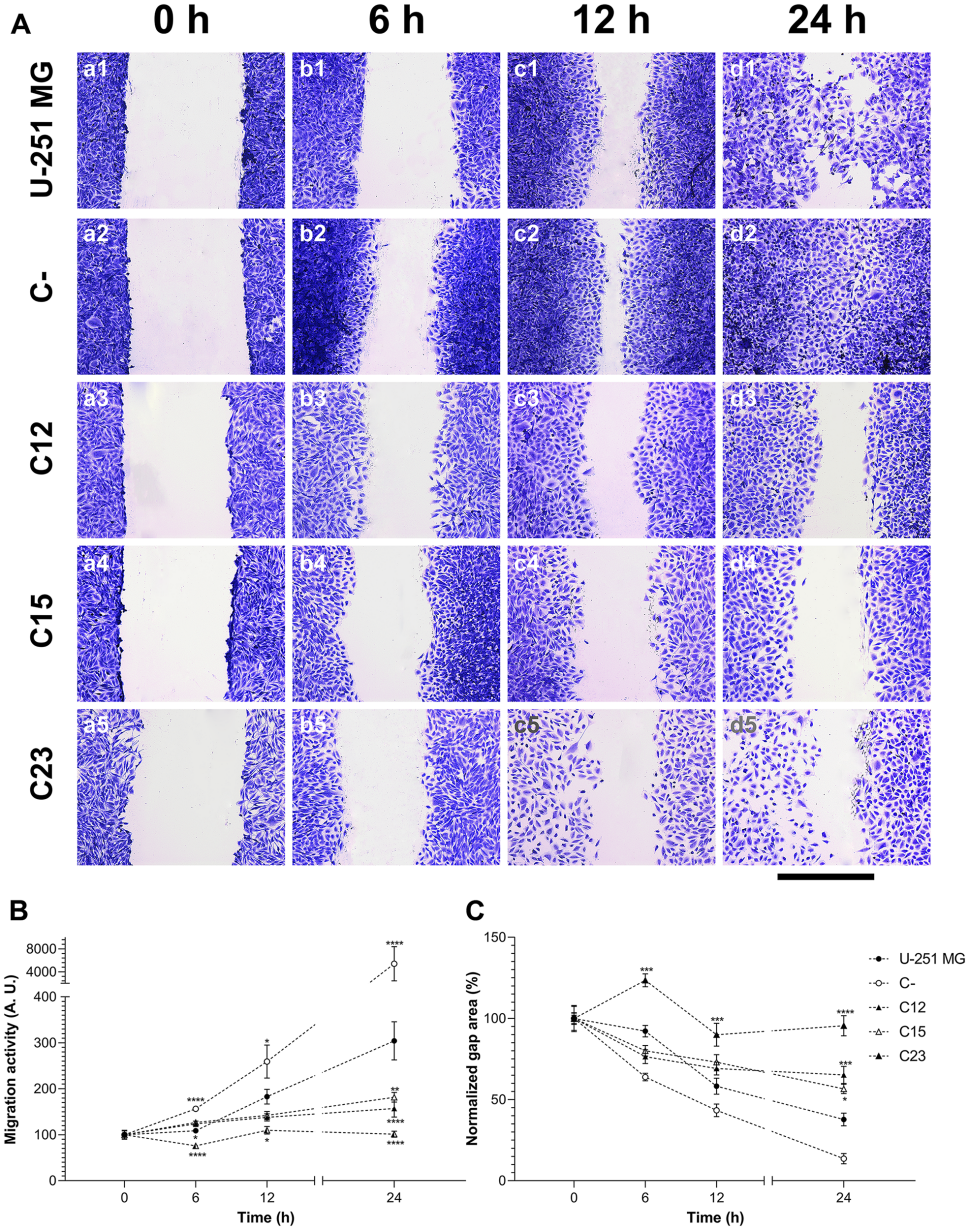
Migration assessment on GBM cells to establish the effects of GPC1. (**A**) U-251 MG (1), C- (2), C12 (3), C15 (4), and C23 (5) cells were submitted to the wound-healing assay to analyze migration patterns of GPC1-silenced cells (C12, C15, and C23). Cells were incubated with 10 µg/ml of mitomycin and stained with crystal violet at 0, 6, 12, and 24 h, after which images were captured. The scale bar refers to 400 µm. (**B**) Migration function and (**C**) normalized gap area were plotted for each cell line, in which the migration function is calculated from the inverse raw gap area. All data are shown as mean ± SEM and were analyzed with the two-way ANOVA with Dunnett’s post hoc test. Statistically significant comparisons are marked as follows: ^*^ *p* < 0.05, ^**^ *p* < 0.01, ^***^ *p* < 0.001, ^****^ *p* < 0.0001 *vs.* U-251 MG.

As previously reported, GPC1 may be essential to cellular migration [24]. We thus proceeded to investigate how this PG would affect GBM cells’ migration pattern with a wound-healing assay (Figure 6A). It was visible that control cells migrated considerably faster than GPC1-silenced clones, measured by the migration function and gap area (Figure 6B and 6C, respectively). The reduction of the gap area would inversely correlate to the migration ability of the cells. Migration was almost completely abolished in C23 cells, with a decrease of 99.3% compared to U-251 MG cells.

**Figure 7:**
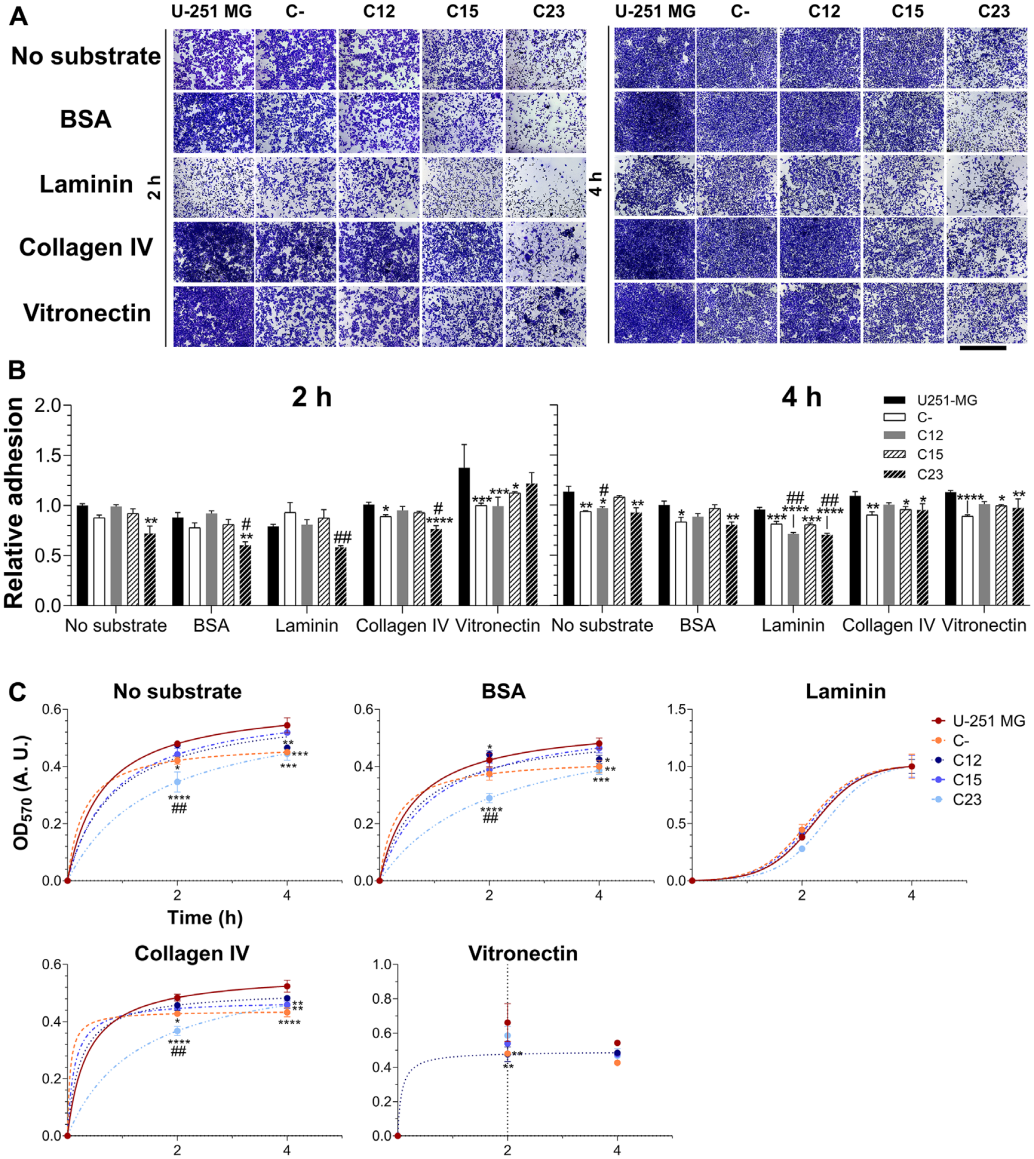
Investigation of GPC1’s role in adhesion of GBM cells. Control GBM cells (U-251 MG and C-) and GPC1-silenced GBM cells (C12, C15, and C23) were assessed for their adhesion properties on laminin, collagen IV and vitronectin, using, as experimental controls, the absence of substrates or BSA blocking of the dish. The experiment was conducted for 2 or 4 h before (**A**) staining with crystal violet and imaging through an optical microscope. The scale bar refers to 1,000 µm. (**B**) The dye was solubilized with 10% acetic acid, and absorbance was measured. The OD_570_ values were normalized to the condition of the absence of a substrate in the original cell line (U-251 MG) at the time of 2 h. (**C**) Otherwise, absorbance values were non-linearly fitted to three-parameter sigmoid models to inspect kinetic models for the adhesion profile for each cell line in each studied condition. Data are shown as mean ± SEM and were analyzed with either two- (in B) or one-way (in C) ANOVA with Dunnett’s post hoc test. Statistically significant comparisons are marked as follows: ^*^ *p* < 0.05, ^**^ *p* < 0.01, ^***^ *p* < 0.001, ^****^ *p* < 0.0001 *vs.* U-251 MG; and ^#^ *p* < 0.05, ^##^ *p* < 0.01 *vs.* C-.

Next, we investigated the adhesion aspects associated with GPC1. Three substrates that are found in the brain and are typically overexpressed in brain tumors were tested: laminin, type IV collagen, and vitronectin [25, 26]. GBM U-251-MG cells display a high affinity with collagen IV and vitronectin, but not with laminin (Figure 7A). There was a reduction in affinity with laminin (Figure 7B) but not with other substrates. The C23 clone did indicate affinity alterations with collagen IV, but this would later appear a result of slower adhesion kinetics in this cell line in particular. When a non-linear, three-parameter sigmoid fitting was performed (Figure 7C), the curves’ parameters (Supplementary Table 2) displayed that GPC1-depleted GBM cell lines, C23 in particular, possessed considerably slower adhesion kinetics with or without substrates. These results reveal that GPC1 is not only vital for GBM cell migration but could also be involved in how fast these cells adhere. GPC1 may also distinctively affect how these tumoral cells interact with laminin.

### GPC1 deficiency sensitizes GBM cells to chemotherapeutic treatment

After verifying that GPC1 influences several biological aspects of GBM, we proceeded to investigate whether this PG would have any consequence on the role of the primary antineoplastic agent used with GBM patients – temozolomide (TMZ).

**Figure 8:**
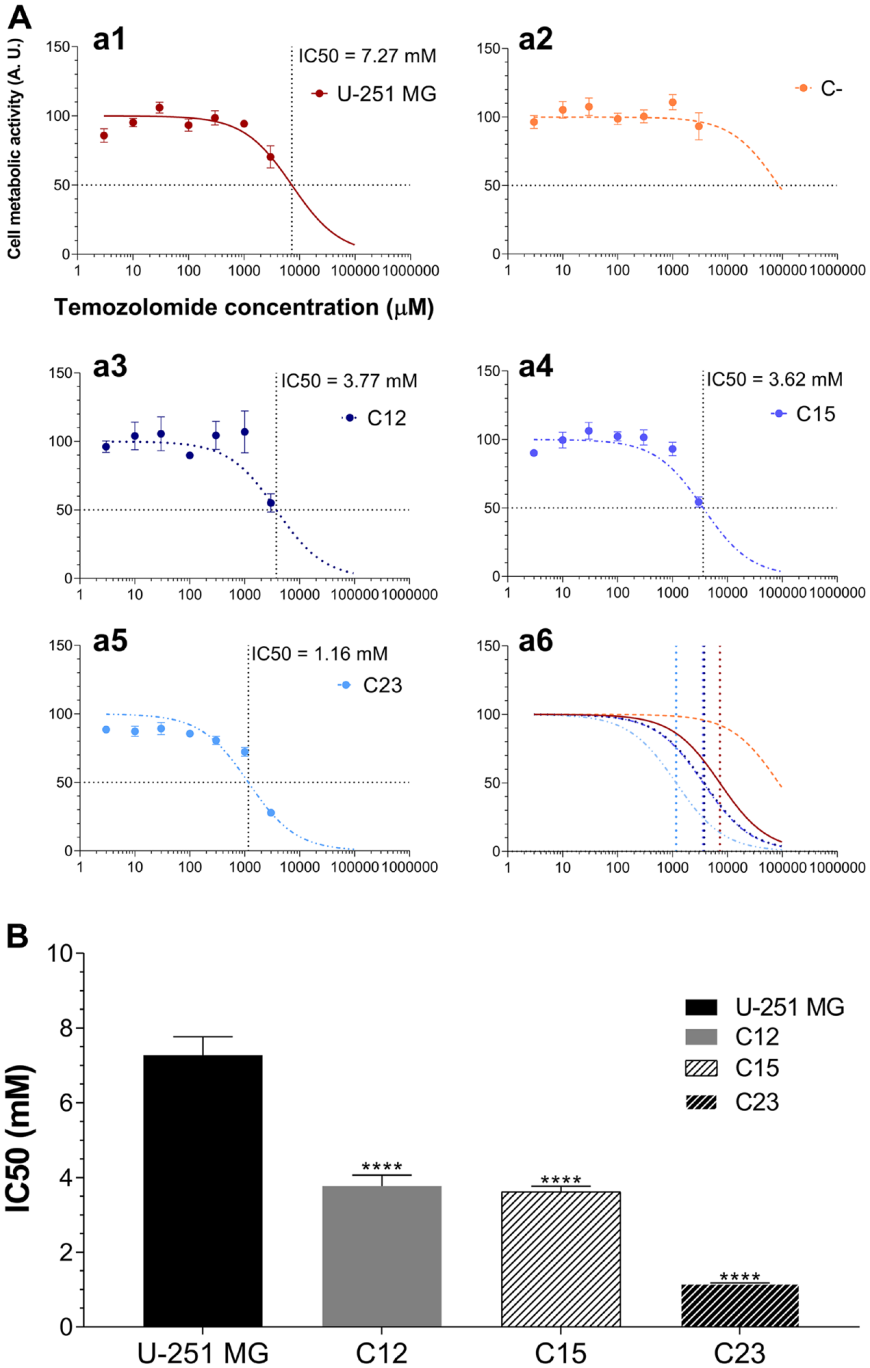
Experiments to investigate whether GPC1 influences TMZ susceptibility in GBM cells. (**A**) U-251 MG (a1), C- (a2), C12 (a3), C15 (a4), and C23 (a5) were incubated with different concentrations of TMZ ranging from 0 – 3 mM for 24 h, and the metabolic activity of the cells was assessed by the MTT assay. Experimental data was non-linear fitted to the Hill equation, the parameters of which are shown in Supplementary Table 3. The horizontal dotted line represents the 50% response and the vertical line the calculated IC50. A representation of all models together is also exhibited (a6). (**B**) Comparison of IC50 values after model adjustment. C-’s IC50 is not shown as the Hill equation could not be adjusted. Data are plotted as mean ± SEM and were analyzed with the one-way ANOVA with Dunnett’s post hoc test. Statistically significant comparisons are marked as follows: ^****^ *p* < 0.0001 *vs.* U-251 MG.

Control cells were not susceptible to TMZ (Figure 8A). While U-251 MG showed an IC50 of (7.27 ± 0.50) mM, C- cells displayed minimal susceptibility to the drug (Figure 8B and Supplementary Table 3). GPC1 knocked-down clones, on the other hand, revealed reduced IC50 levels; this variable in C23 cells was more than six times lower than in U-251 MG. These data indicate that GPC1 may integrate the resistance mechanism of this alkylating drug and could be a potential target for future therapies for GBM.

## DISCUSSION

Even though HSPGs have been shown to play central roles in cancer, few studies have linked their effects to GBM [27]. Our hypothesis was traced from a handful of works that indicated a probable central role of GPC1 in these tumors [19, 20]. After generating GBM GPC1-silenced clones and selecting which were considered the most suitable ones, several experiments were performed in an attempt to trace different cellular behavioral patterns associated with GPC1 presence or absence.

The transcriptional profiles of selected HSPGs, β-catenin and MMP9 were affected due to GPC1 in GBM cells. The heparanome is a sophisticated machine in which a plethora of enzymes and their many isoforms not only synthesize HS but modify these GAG chains; this, in turn, creates an intricate puzzle containing negative and positive feedback loops that may be affected by altering the expression of a single component [28]. This has been demonstrated with SDC1 in malignant mesothelioma [29], and here we may be detecting similar effects on other HSPGs by simply modulating GPC1. Moreover, in downregulating GPC1, β-catenin expression was reduced, which may indicate a reduction in canonical Wnt signaling. As previously demonstrated for esophageal squamous cell carcinoma, GPC1 does indeed directly affect β-catenin expression [30]. Similar effects have been reported for GPC3, -4 and -5 in hepatocellular, pancreatic, and lung cancers. Although this effect has never been demonstrated for GPC1 in GBM, we could not show which possible Wnt ligand would associate directly with the PG in our focus. Regarding the MMPs, we were able to verify that GPC1 was positively correlated with MMP expression, which may reveal a role of the PG in GBM invasiveness. It has been suggested that MMP9 could bind to GPCs and possibly with GPC1 [17, 31]. Nevertheless, there is no data on whether GPCs could influence MMP2 expression nor a more thorough description of how these expressions would be altered in different kinds of cancer, such as GBM.

Cellular proliferation and, consequently, tumor growth are critical characteristics of GBM [32]. Thus, our primary focus was to assess whether GPC1 would modulate either cellular growth or proliferation patterns in GBM cells. As predicted, GPC1-silenced cells not only grew significantly slower but also were more prone to remain in a non-proliferating state, as shown in the proliferation and clonogenicity assays. When cell cycle phases were considered, significant differences were mainly found between the G1 and G0 phases, in which GPC1-downregulated cells would primarily rest in the G0 phase. The scientific literature is robust regarding how either GPC1 or other GPCs positively regulate cellular proliferation in pancreatic cancer, esophageal squamous cell carcinoma, rhabdomyosarcoma, hepatocellular carcinoma, and liver cancer [30, 33–37]. However, there have never been data on the GPC1 link to the proliferation of GBM.

Subsequently, we investigated not only GPC1 localization in GBM cells, its regionalization in lipid rafts, and its association with SDC4 and GPC3; we also investigated whether the downregulation of GPC1 would influence its ability to interact with those molecules. SDC4 and GPC1 are believed to play together a fundamental role in myoblast proliferation, differentiation and migration during myogenesis by mediating the signaling of fibroblast growth factor 2 [22]. GPC1 and its isoform GPC3, on the other hand, have been shown to colocalize and together interact with bone morphogen protein 2 in mesenchymal cells with an osteoprogenitor phenotype [23]. From our results, it was evident that GPC1 intensely labeled GBM control cells in the cellular membrane. Nevertheless, staining in EVs and during their interaction with the cellular membrane could also be identified. Recently, several scientific studies have been considering GPC1 a possible biomarker in pancreatic ductal adenocarcinoma and colorectal cancer through its detection in patients’ serum exosomes [38–45]. We have first uncovered how EVs containing GPC1 are also enriched in GBM. We could not establish a precise link between GPC1 localization in lipid rafts and GBM, although it was, indeed, associated with SDC4 and GPC3. It has been previously demonstrated that GPC1 may be localized in lipid rafts of neuroblastoma cells or myoblasts [21, 46]; however, there were no previous data of this PG in GBM cells. On the other hand, previous reports have identified GPC3 outside of these microdomains [47]. We did observe a more profound association between GPC1 and -3 than that of GPC1 and FLOT1, indicating that our focused PG could, perhaps, be also localized outside of lipid rafts and interact, for instance, with other molecules such as GPC3. As observed, the relationship between these GPCs was also observed in EVs, and recently, GPC3-containing exosomes in cancer have been described in the literature [48]. Moreover, SDC4 presence in membrane rafts is controversial; there have been reports explicitly placing this HSPG in these microdomains, or not associated with them at all [21, 49]. In our context, we did find GPC1 close to SDC4, specifically in cell-cell junctions, in which SDC4 have been previously described [50]. Curiously, SDC4 protein levels revealed to remain constant throughout all tested cell lines, including the ones silenced for GPC1, which contrasted with the gene expression results, that revealed that SDC4 could be downregulated when the expression of GPC1 was low. This effect could be related to PG’s low or almost absent turnover, especially in the skin, cartilage, lung and muscles [51]. Although our data may indicate the actions of GPC1 outside of lipid rafts in association with either SDC4 or GPC3 in GBM, it is evident that further investigations are needed to confirm that GPC1 is differently located in these specific tumors.

We then proceeded with our investigation on the GPC1 influence on GBM cellular migration and adhesion. Our data suggested a fundamental role of this PG in tumoral cells’ migration. When GPC1 was silenced, migration activity was highly reduced or almost abolished, as seen in the C23 clones. These results are of great interest as migration is one of the primary factors associated with the proliferation of GBM [52]. Although there is no data regarding GPC1’s role in GBM cellular migration, previous studies have lent support to how essential this PG is in this biological role both in pathological (i. e., cancer) or physiological conditions [24, 53]. Other isoforms, such as GPC3, -4 and -5, have been shown to interact with either Wnt ligands or growth factors and to mediate the migration of tumoral cells in breast, lung, and hepatocellular cancers [54–59]. The ability of either Wnt signaling or several growth factors signaling to influence cellular motility is well known [60, 61]. Therefore, we suggest that GPC1 is crucial for cell motility in GBM, as the PG may bind proteins intrinsically linked with migration.

GPC1 did also reveal effects on cellular adhesion. Our data indicate that GPC1 may mediate interaction with laminin in this *in vitro* model. Additionally, adhesion kinetics was substantially affected, particularly in the C23 clone, impinging alterations on collagen IV binding intensity. Laminins are a predominant family of proteins in the ECM that is tightly involved in angiogenesis, invasiveness, stemness, and metastasis in cancer [62]. Collagen IV is expressed in the ECM and basement membrane of several tissues, including the brain [63]. In gliomas, both laminins and collagen IV are up-regulated as well as their corresponding integrin receptors [64–66]. Moreover, HSPGs are pivotal mediators in cell adhesion. It is clear that SDCs do relate much more to this biological aspect than GPCs. SDCs, specifically SDC4, and not GPCs, are the main components for the formation of stable focal adhesions on fibronectin, interacting with focal adhesion kinase (FAK) or binding to ADAM 12 in order to assemble complexes with integrin receptors [67–70]. Nevertheless, it has been previously assessed that GPC1 binding to α_4_ chains of collagen is crucial for Schwann cell adhesion, yet no effects were obtained with collagen IV [71]. Indeed, Liu and collaborators did consider that SDCs would be more prone to interact with collagens than GPC1 [72]. These observations could point to the altered kinetics presented, as opposed to a direct interaction between collagen IV and GPC1. It was evident that GPC1-depleted cells possessed slower adhesion kinetics, and as far as the literature can support, an interaction between ligand (the substrates) and receptors (integrins) should be altered in a manner to hinder cellular response [73, 74]. Therefore, we hypothesize that GPC1 depletion may dampen distinct integrin receptors and synthesis of ECM components inhibiting cellular adhesion. We could not trace a parallel between our findings with GPC1 affinity to laminin in the scientific literature, much less cross-reference the data with GBM profiles. These data support indications of GPC1-laminin interactions and how they affect GBM tumoral biology; however, further investigation is necessary to prove the protein-protein relation between the PG and this substrate or its receptor.

Lastly, we were compelled to correlate GPC1 with TMZ susceptibility in GBM cells. GBM cells revealed the need for high concentrations of TMZ to promote cell death when in elevated confluence. U-251 MG cells had an IC50 of (7.27 ± 0.50) mM, and C-’s IC50 could not be calculated as a result of limited response to TMZ. GPC1-silenced cells had IC50 from two to six times lower than the tested control cell lines. TMZ is a prodrug that spontaneously converts itself to MTIC, a methylating agent, that can transfer methyl groups, in particular, to the DNA, resulting in errors in the DNA mismatch repair (MMR) system. The accumulation of DNA nicks inhibits proliferation by blocking the G2/M cell cycle boundary [75, 76]. Our data reaches a contradiction in this experiment, as TMZ needs cells in a high-proliferation state to induce DNA damage, and resting cells, such as GPC1-silenced clones, are seen as a protective mechanism to the genomic alterations caused by the alkylating agent [77]. Nonetheless, GBM cells may present varying degrees of resistance to alkylating agents through the expression of O^6^-methylguanine-DNA-methyltransferase (MGMT) or demethylation of this enzyme’s promoter [78, 79]. The scientific literature is practically absent of information regarding proteoglycan and pharmacological resistance in cancer, but Saito and colleagues did demonstrate that GBMs with high contents of GPC1 would be positively correlated to MGMT expression [19]. Additionally, GAGs, themselves, may interact with various drugs through electrostatic forces, hydrophobic interactions and the carbohydrate conformational changes, including antineoplastic agents [80–82]; and have their synthesis affected through drug-mediated mechanisms [83]. We cannot know precisely how TMZ may affect GAG synthesis or interact with them as there are no reports in the literature regarding this information. Nonetheless, we believe that these data could indicate the first basis on how TMZ, or possibly other alkylating agents, may be involved with GAG, especially HS, either by interacting with them or affecting their synthesis. Moreover, we present indications that GPC1 may possibly be associated with resistance mechanisms of alkylating agents in GBM; thus, explaining why in the downregulating panorama of this PG, GBM cells were much more susceptible to the antineoplastic agent.

This study aimed to investigate whether GPC1 could modulate GBM tumoral behavior. Our findings concluded that this GPC is a pivotal macromolecule in this environment, inciting neoplastic growth, proliferation, migration, adhesion, and chemotherapy resistance. By downregulating the expression of GPC1, the tumoral profile was severely hindered, indicating that this GPC is a vital element in GBM tumorigenesis.

## MATERIALS AND METHODS

### Cell culture and ethics disclaimer

U373-MG (Uppsala, Sigma 08061901) cells were purchased from Sigma-Aldrich (Saint Louis, MO, USA) while U-251 MG (Sigma 09063001) and T98G (ATCC^®^ CRL-1690™) cells were donated for this work. U373-MG cells were cultivated in MEM supplemented with 10% of FBS and 100 µg/mL of penicillin and streptomycin (Gibco^®^). U-251 MG and T98G cell lines were kept in DMEM with the same supplementation mentioned above. The cells were grown at 37°C in a humidified atmosphere with 5% CO_2_.

This work was approved by the Institutional Ethics Committee of the Universidade Federal de Sao Paulo (protocol number: 3023170219).

### Glypican-1 knock-down and cell cloning

U-251 MG cells had the GPC1 gene silenced using the SMARTvector Human Lentiviral GPC1 shRNA (Dharmacon; Lafayette, CO, USA). All shRNA sequences used are shown in Supplementary Table 4. In brief, lentivectors for different MOI evaluations (10, 20, and 30) were diluted in DMEM containing polybrene to a final concentration of 2 µg/mL (Merck; Darmstadt, Germany). Cells were harvested, and 5 × 10^4^ cells were added for each 500 µL of the lentivector solution. Lentivectors without an annealing sequence (negative control), cells untreated with lentivectors (double negative control), and shRNA for GAPDH (positive control) were also employed. After 72 h, the medium was changed to complete DMEM supplemented with 2 µg/mL of puromycin for the selection of infected cells. Polyclonal, puromycin resistant cell lines were obtained and characterized by RT-qPCR.

For the generation of monoclonal cell lines, the most suitable polyclonal cell line was selected and grown in 100-mm dishes. After 80% confluence was present, the conditioned medium was collected and centrifuged at 1000×*g* for 5 min, after which the supernatant was collected. The plated cells were detached and diluted to 5 cells/mL in the conditioned medium. The cells were plated in a 96-well plate with 100 µL in each well (0.5 cell per well). After one week, wells containing single colonies were further examined. After 50% confluence was observed, colonies were expanded, generating their own monoclonal cell lines. This experiment produced 20 viable monoclonal GPC1-silenced cell lines.

### RNA extraction and real-time quantitative PCR

All tested cells, including controls and generated clones, were plated on 60-mm dishes. After reaching 80% confluence, the medium was removed, and 1 mL of Trizol (Invitrogen™) was added and incubated for 5 min. RNA was extracted using the Direct-zol RNA purification kit (Zymo Research; Irvine, CA, USA), and cDNA was obtained from 300 ng of RNA template, in a reverse transcription reaction of 20 µL, containing 3 mM of MgCl_2_, 500 µM of dNTP mix, 80 U of ImProm II Reverse Transcriptase (Promega; Madison, WI, USA), 20 U of RiboLock RNase Inhibitor and 500 nM of oligo (dT)_15_ primer (Promega) in ImProm II Reverse Transcriptase 1× buffer (Promega). The reaction was conducted at 25°C for 5 min for primer annealing, 42°C for 60 min for transcriptase activity and at 70°C for 10 min for inactivation.

Real-time PCR was performed in 10 µL reactions, in which 100 ng of cDNA were mixed with 5 µL of PowerUp™ SYBR^®^ Green Master Mix (Thermo Fisher Scientific) and 500 nM of specific forward/reverse primer pairs, as listed in Supplementary Table 5. qPCR reaction was conducted in 7500 Real-Time PCR System (Applied Biosystems^®^; Foster City, CA, USA).

### Immunofluorescence and confocal imaging

U-251 MG, C-, C12, C15, and C23 cells were harvested, and 2 × 10^4^ cells were plated in 24-well plates coated with 13 mm borosilicate coverslips. After 48 h, the medium was drained, and wells were washed with PBS. Cells were then fixed with 4% paraformaldehyde for 15 min at room temperature. After washing with PBS and a 0.1 M glycine solution, cells were blocked for 1 h with 3% BSA in PBS and incubated with primary antibodies at 1:200 in 1% BSA overnight at 4 °C. Antibodies used in this experiment were anti-GPC1 (goat, R&D, AF4519), anti-GPC3 (mouse, R&D, MAB2219), anti-GFAP (rabbit, Millipore, AB5804), anti-SDC4 (rabbit, Santa Cruz, sc-15350), and anti-flotillin-1 (mouse, Novus, NBP1-87498). The wells were then washed with PBS and incubated with the secondary antibodies at 1:250 in 1% BSA for 1 h at room temperature, in which anti-goat 488 (A11055), anti-mouse 647 (A31571) and anti-rabbit 647 (A31573; all Thermo Fisher Scientific) were used for labeling. Cells were stained with DAPI at 2.5 µg/mL in 0.05% saponin for 15 min and further washed with PBS. Subsequentially, coverslips were rinsed in water and mounted in Fluoromount™ (SouthernBiotech; Birmingham, AL, USA) in clean slides. Slides were imaged in Leica Confocal Microscope TCS SP8 CARS.

### Cell metabolic activity assay

U-251 MG, C-, C12, C15, and C23 cells were harvested, and 3,000 cells were plated in 96 well plates. After 24, 48, 72 and 96 h, MTT at 5 mg/mL was added and incubated for 4 h. The medium was then removed, and the formazan precipitate was solubilized with DMSO. The plates were agitated, and wells had their absorbance read at 570 nm (OD_570_). Blank wells were subtracted, and data were normalized from wells in which MTT was absent.

### Cell proliferation and cell cycle profile analysis

For the cell-proliferation assay, U-251 MG, C-, C12, C15, and C23 cells were harvested, and 1 × 10^4^ cells were plated in 24-well plates coated with 13 mm borosilicate coverslips. After 24 h, the complete medium was replaced with a serum-deprived medium, which was cultivated for 6 h before being again substituted with complete medium. After 48 h, the proliferation assay was performed as mentioned previously with minor modifications [84]. The medium was drained, and wells were washed with PBS. Cells were then fixed with 4% paraformaldehyde for 15 min at room temperature. After washing with PBS and a 0.1 M glycine solution, cells were permeabilized in 0.2% Triton X-100 in PBS for 15 min and then blocked in 5% FBS with 0.1% TWEEN^®^ 20 for 30 min. Cells were then incubated with anti-Ki-67 antibody (mouse, 556003, BD Biosciences) at 1:200 in blocking buffer overnight at 4°C. The wells were then washed with PBST (0.1% TWEEN^®^ 20) and incubated with the anti-mouse 647 at 1:250 in PBST for 1 h at room temperature. Cells were stained with DAPI at 2.5 µg/mL in PBST for 15 min and further washed with PBS. Subsequently, coverslips were rinsed in water and mounted in Fluoromount™ on clean slides. Slides were imaged using a Leica TCS SP8 CARS Confocal Microscope (Leica Microsystems; Wetzlar, Germany).

For cell cycle analysis, cell lines were plated in 100-mm dishes, and after 24 h were serum-deprived for 6 h. After reaching 80% confluence, cells were detached with 500 µM of EDTA and collected by centrifugation (3,000 rpm for 3 min). Cells were then fixed in cold 70% ethanol while in agitation and left at –20°C for at least 2 h. Cells were washed twice in staining buffer (1% FBS, 0.09% NaN_3_ in PBS) and collected in 5-mL tubes. Cells were resuspended in 500 µL of staining buffer, and to each tube, 10 µL of a solution containing 50 µg/mL of propidium iodide (PI, Thermo Fischer Scientific) and 100 µg/mL of RNase A (Sigma-Aldrich) was added. The tubes were left on ice for at least 30 min before assessment in a BD Biosciences FACSCalibur cytometer (BD Biosciences; Franklin Lakes, NJ, USA).

### Clonogenicity assay

U-251 MG, C-, C12, C15, and C23 cells were harvested, and 400 cells were plated in 6 well plates for 8 days. The wells were washed twice with cold PBS, and cells were fixed with cold methanol for 10 min. The content was stained with 0.5% crystal violet (m/v) in 25% (v/v) methanol for 10 min in the dark, then destained with type I water. After drying overnight, colonies were assessed for formations with more than 50 cells present [85].

### Cell migration assessment

U-251 MG, C-, C12, C15, and C23 cells were harvested, and 4.5 × 10^4^ cells were plated in 24 well plates for 48 h. A linear scratch was made with a pipette tip of 200 µL, and the wells were washed with complete medium once. The cells were then incubated in medium supplemented with 10 µg/mL of mitomycin, and after 0, 6, 12, and 24 h the gap was imaged, and its area was quantified using the ImageJ software.

### Cell adhesion investigation on matrix substrates

Laminin and collagen IV, both at 20 µg/mL, and vitronectin at 5 µg/mL (Sigma Aldrich) were added to 96 well plates and left for 1 h at room temperature. The content was then drained, and wells were blocked with 1% BSA for 30 min at 37°C. The plates were washed with sterile PBS, and 2 × 10^4^ cells of U-251 MG, C-, C12, C15 or C23 cells were added to the wells and incubated for 2 or 4 h. The cells were then washed twice with PBS, fixed with methanol, stained with crystal violet, and destained with type I water. After drying, the wells were imaged, and the dye was solubilized with 10% acetic acid for absorbance quantification at 570 nm.

### Temozolomide pharmacological profiles

U-251 MG, C-, C12, C15, and C23 cells were harvested, and 2 × 10^4^ cells were plated in each well of a 96-well plate. After 24 h, the medium was replaced by variable concentrations (eight points ranging from 0–3 mM) temozolomide (Sigma Aldrich). After an additional 24 h, the MTT metabolic activity assay was performed as previously described, in which OD_570_ values were normalized to untreated cells’ absorbance.

### Statistical analysis

Data were analyzed using GraphPad Prism 8.0, Microsoft Office Excel 2016, and Minitab 18. Two- and one-way analysis of variance (ANOVA) was used, combined with Dunnett’s test, for comparison to control samples; Bonferroni’s test was used for particular contextual analysis. For model assessment, experimental data was either linear or non-linear fitted according to the least-squares fitting method. Statistical significance was considered when *p* < 0.05.

## SUPPLEMENTARY MATERIALS



## Abbreviations

ANOVA: Analysis of variance
BSA: Bovine serum albumin
CNS: Central nervous system
EV: Extracellular vesicle
FAK: Focal adhesion kinase
FGF: Fibroblast growth factor
FLOT1: Flotillin 1
GAG: Glycosaminoglycan
GAPDH: Glyceraldehyde-3-phosphate dehydrogenase
GBM: Glioblastoma
GPC: Glypican (and isoforms)
GPI: Glycosylphosphatidylinositol
HS: Heparan sulfate
HSPG: Heparan sulfate proteoglycan
MGMT: O6-methylguanine-DNA-methyltransferase
MMP: Metalloprotease
MMR: Mismatch repair
MOI: Multiplicity of infection
OD570: Optic density at 570 nm
PBS: Phosphate buffered saline
PCC: Pearson correlation coefficient
PG: Proteoglycan
PI: Propidium iodide
PPIB: peptidyl-prolyl cis-trans isomerase B
SDC: Syndecan (and isoforms)
TMZ: Temozolomide
WHO: World Health Organization
