# Supplementary material for: Glypican-1 in human glioblastoma: implications in tumorigenesis and chemotherapy
Source: Oncotarget. 2020 Mar 3;11(9):828–45. doi: 10.18632/oncotarget.27492 (PMC7061737; doi:10.18632/oncotarget.27492)
Supplement: Supplementary file 1 [file oncotarget-11-828-s001.pdf]

Glypican-1 in human glioblastoma: implications in tumorigenesis and chemotherapy

SUPPLEMENTARY MATERIALS

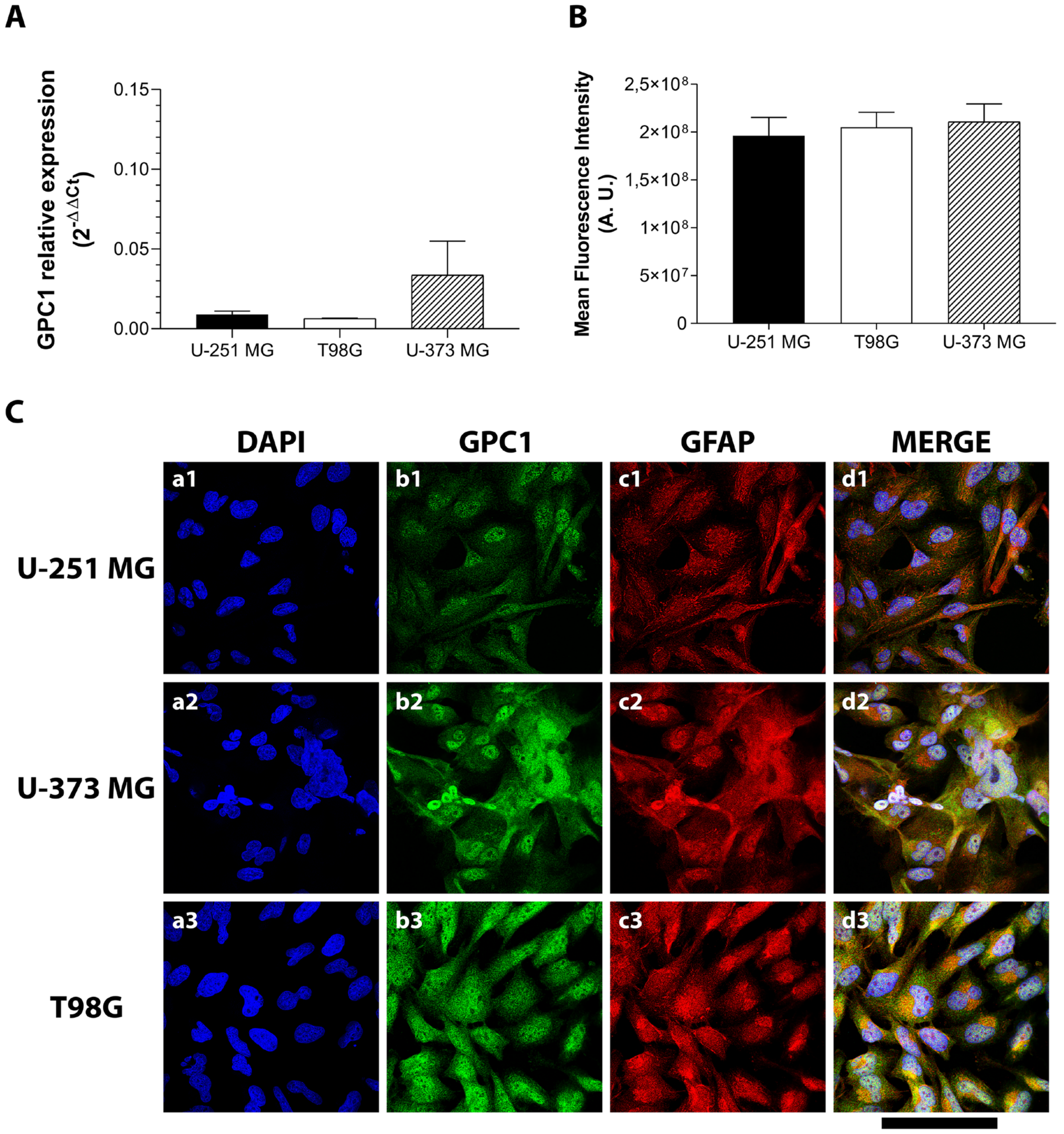

Supplementary Figure 1: GPC1 gene and protein analysis on three selected GBM cell lines. (**A**) The relative expression of GPC1 to GAPDH and β-actin was assessed by RT-qPCR in U-251 MG, T98G and U-373 MG cell lines, in which n = 5–6. (**B**) Immunofluorescence assay staining of GPC1 was quantified in two to three fields in three independent assays (n = 8) and expressed as mean fluorescence intensity for each investigated cell line. All data are represented as mean ± SEM. The one-way ANOVA with Tukey's pos*t*-test was performed to investigate sample differences, and none were identified. (**C**) GBM U-251 MG (1), U-373 MG (2) and T98G (3) cells were analyzed for GPC1 (b) localization against GFAP (c). The cells' nuclei were also stained with DAPI (a), and a composite is shown (d). The scale bar indicates 100 µm.

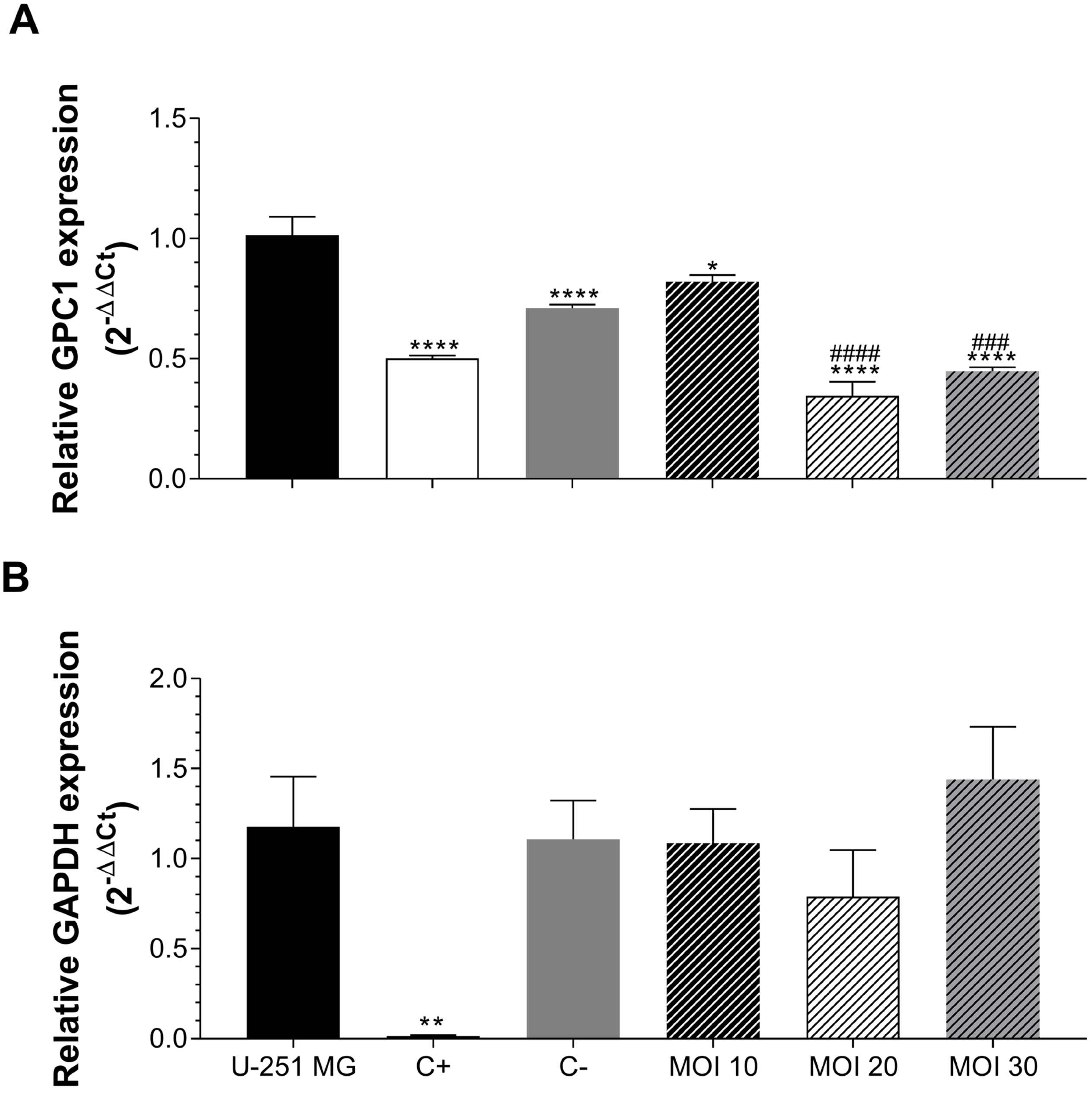

Supplementary Figure 2: Polyclonal cell lines gene silencing results for U-251 MG. The lentiviral infection was performed in three multiplicities of infection (MOI) for GPC1 shRNA, and GPC1 and GAPDH were quantified by RT-qPCR in relation to β-actin and peptidyl-prolyl cis-trans isomerase B (PPIB) and related to U-251 MG as control. (**A**) GPC1 gene expression in control GBM cell lines (U-251 MG, C+, and C-) and GPC1-silenced polyclonal cell lines (MOI 10, MOI 20, and MOI 30) was performed to verify which polyclonal cell line displayed more suitable GPC1 silencing. (**B**) GAPDH gene expression was also investigated to validate the experiment through C+. All data are represented as mean ± SEM. The one-way ANOVA with Dunnett's pos*t*-test was performed, and significant differences are marked as follows: $^{*}p < 0.05$; $^{**}p < 0.01$; $^{****}p < 0.0001$ *vs.* U-251 MG and; $^{###}p < 0.001$; $^{####}p < 0.0001$ *vs.* C-.

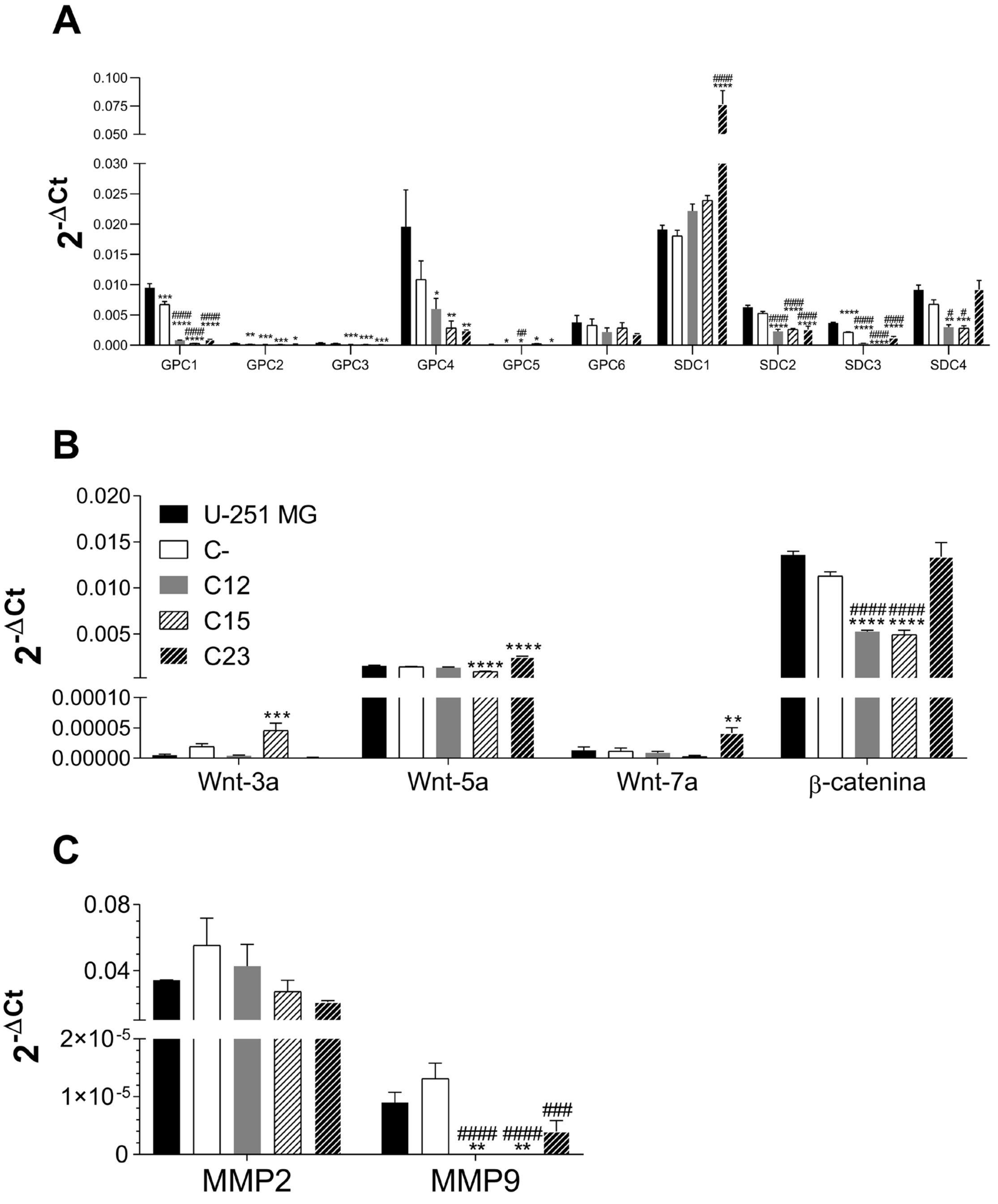

Supplementary Figure 3: Expression profiles of control and GPC1 knock-down GBM cell lines of HSPG, selected Wnt ligands and MMPs in relation to β-actin. Control GBM cell lines (U-251 MG and C-) and GPC1-silenced clones (C12, C15, and C23) were analyzed by RT-qPCR in relation to β-actin in order to verify the expression of analyzed genes (**A**) membrane-bound HSPGs, (**B**) selected Wnt ligands or β-catenin, or (**C**) selected MMPs; were mainly expressed in these tumoral cells. Data are plotted as mean ± SEM and n = 5–10. The two-way ANOVA with Dunnett's post-hoc test was performed, and statistically significant comparisons are coded as follows: $^{*}p < 0.05$, $^{**}p < 0.01$, $^{***}p < 0.001$, $^{****}p < 0.0001$ *vs.* U-251 MG; and $^{\#}p < 0.05$, $^{\#\#\#}p < 0.001$, $^{\#\#\#\#}p < 0.0001$ *vs.* C-.

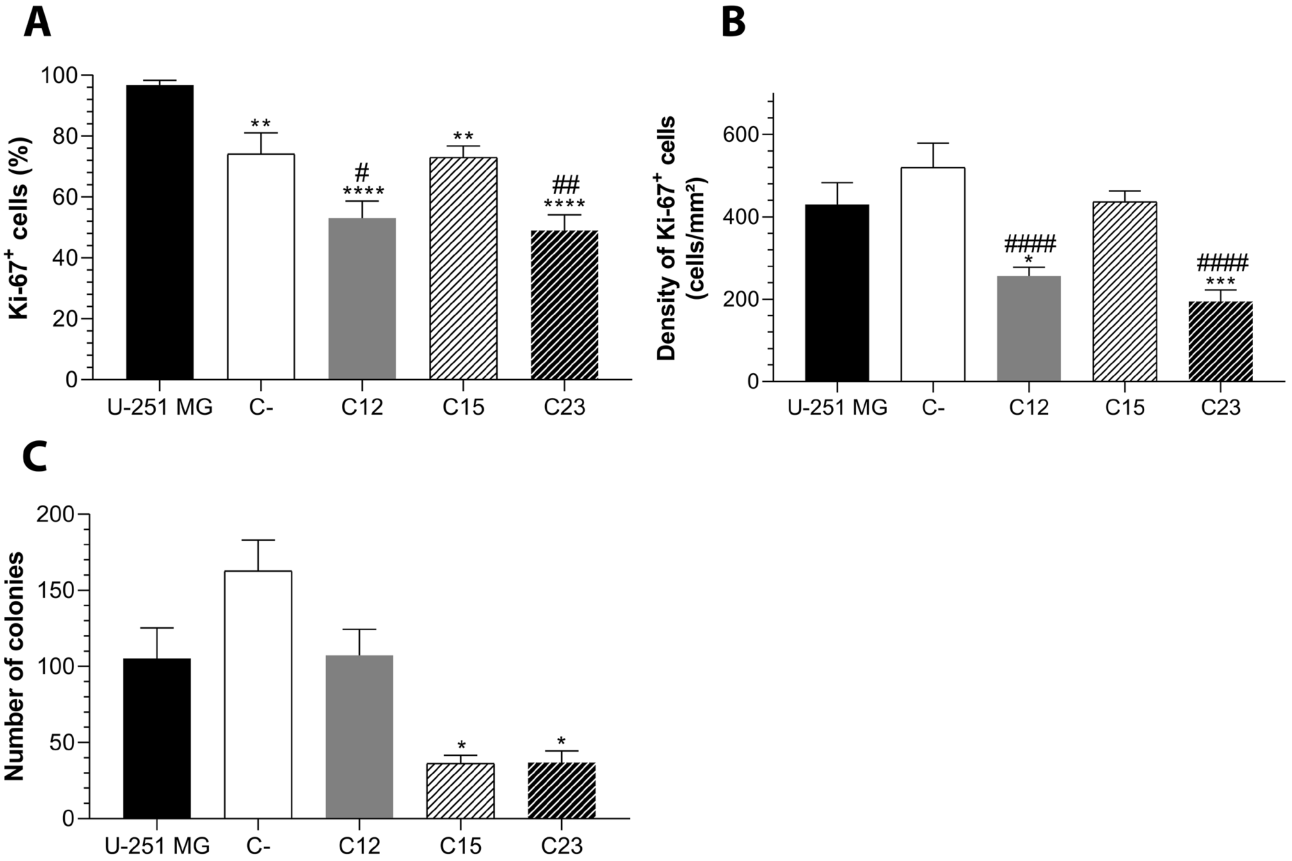

Supplementary Figure 4: Quantification of proliferation and clonogenicity assays to evaluate GPC1's role in these biological processes in GBM. Control GBM cells (U-251 MG and C-) and GPC1-silenced clones (C12, C15, and C23) were stained for Ki-67^{+}, and the presence of the antigen in cells' nuclei indicated proliferating cells. (**A**) The fraction of Ki-67^{+} (%) was plotted, or (**B**) cells were counted in an area to obtain Ki-67^{+} cell density. (**C**) For assessment of the clonogenic potential, cells were left to grow, and colonies containing at least 50 cells were quantified. Data are represented as mean ± SEM. The one-way ANOVA with Dunnett's pos*t*-test was performed, and significant differences are marked as follows: $^{*}p < 0.05$; $^{**}p < 0.01$; $^{****}p < 0.0001$ *vs.* U-251 MG and; $^{\#}p < 0.05$, $^{\#\#}p < 0.01$; $^{\#\#\#\#}p < 0.0001$ *vs.* C-. The sample size was $n = 12$ for the proliferation assay and $n = 4$–6 for the clonogenicity assay.

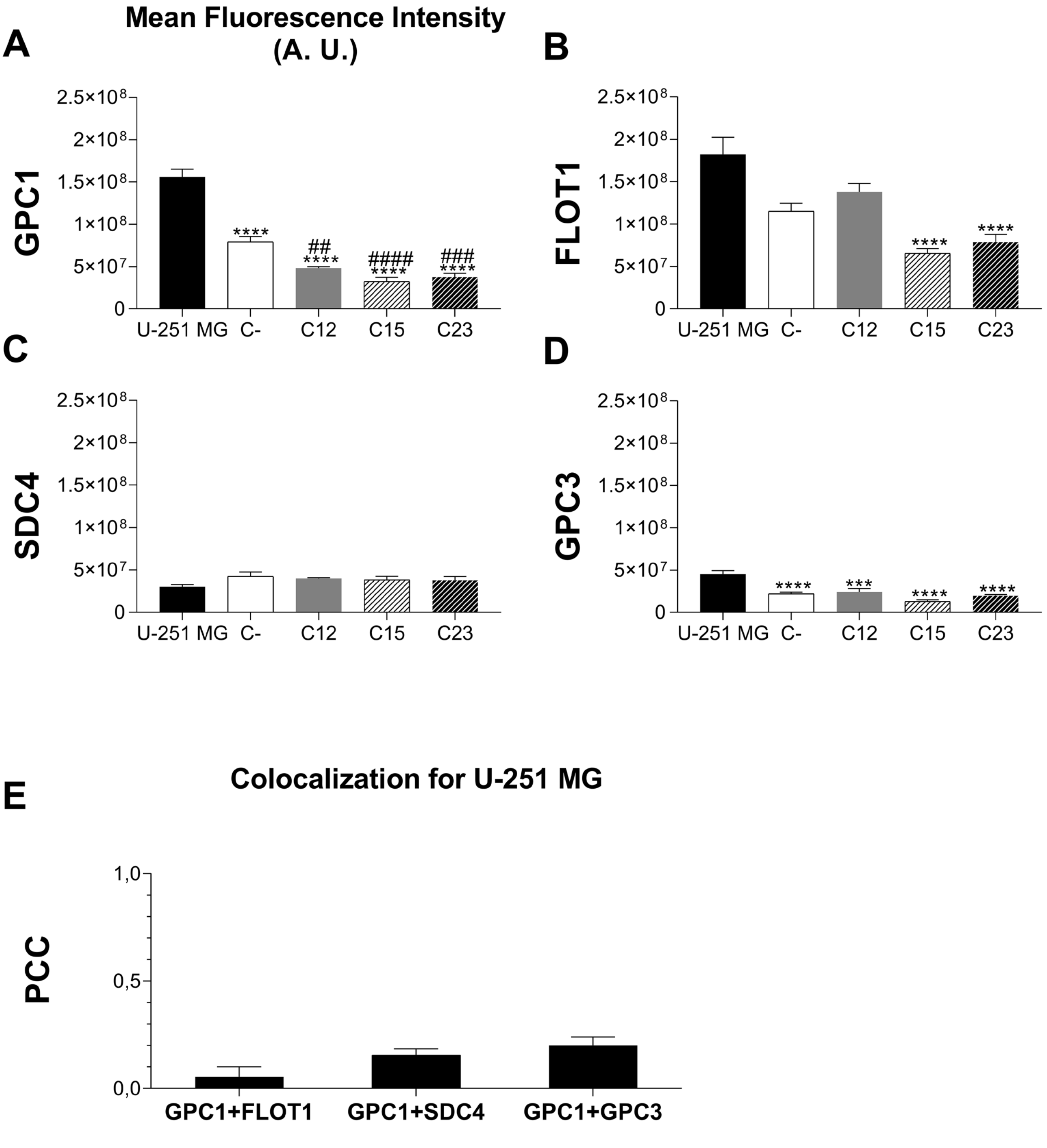

Supplementary Figure 5: Quantification of immunofluorescence assays in GBM control cells and GPC1 knock-down clones. U-251 MG, C-, C12, C15, and C23 cells investigated regarding GPC1, FLOT1, SDC4 and GPC3 by immunofluorescence and confocal microscopy. (**A**) GPC1 mean fluorescence was quantified in all used cell lines. (**B**) FLOT1 was used to evaluate the GPC1 association with lipid rafts. (**C**) SDC4 and (**D**) GPC3 are two other HSPGs commonly associated with GPC1. (**E**) Represents the Pearson correlation coefficient (PCC) that symbolizes the degree of colocalization between GPC1 and FLOT1, SDC4 or GPC3 in U-251 MG cells; All data are represented as mean ± SEM. The one-way ANOVA with Dunnett's pos*t*-test was performed, and significant differences are marked as follows: $^{***}p < 0.001$; $^{****}p < 0.0001$ *vs.* U-251 MG and; $^{\#\#}p < 0.01$, $^{\#\#\#}p < 0.001$; $^{\#\#\#\#}p < 0.0001$ *vs.* C-. The sample size was n = 6 in all experiments.

Supplementary Table 1: Linear regression parameters for the growth curves adjusted to the viability assay

Cell line	Growth rate (h^{-1})	*y*-intercept	$R^2_{adj.}$
U-251 MG	26.94 ± 2.18	166.8 ± 143.0	0.735
C-	17.71 ± 1.16	242.4 ± 76.41	0.808
C12	12.58 ± 1.27	395.5 ± 83.2	0.640
C15	10.76 ± 0.94	87.0 ± 61.9	0.702
C23	7.67 ± 0.85	121.7 ± 56.1	0.592

The growth rate is obtained from the curve inclination. The goodness of fit is expressed by the adjusted correlation coefficient (R^2_{adj}).

Supplementary Table 2: Non-linear regression parameters of kinetic adhesion models for GBM control or GPC1-silenced cell lines

	Cell	Ad_{max} (U. A.)	K_{Ad} (h)	Ad_{min} (U. A.)	$R^2_{adj.}$
No substrate	U-251 MG	0.629 ± 0.051	0.622 ± 0.269	0.000 ± 0.016	0.984
	C-	0.484 ± 0.021	0.300 ± 0.124	0.000 ± 0.008	0.995
	C12	0.496 ± 0.020	0.150 ± 0.000	0.000 ± 0.009	0.995
	C15	0.608 ± 0.021	0.850 ± 0.000	0.007 ± 0.021	0.968
	C23	0.621 ± 0.124	1.593 ± 0.912	0.000 ± 0.024	0.943
BSA	U-251 MG	0.557 ± 0.055	0.638 ± 0.332	0.000 ± 0.017	0.977
	C-	0.430 ± 0.048	0.300 ± 0.324	0.000 ± 0.018	0.966
	C12	0.536 ± 0.077	0.760 ± 0.509	0.006 ± 0.022	0.956
	C15	0.578 ± 0.065	0.973 ± 0.424	0.000 ± 0.017	0.975
	C23	0.579 ± 0.076	2.002 ± 0.664	0.000 ± 0.013	0.979
Laminin	U-251 MG	1.017 ± 0.017	2.217 ± 0.037	-0.006 ± 0.016	0.995
	C-	1.013 ± 0.070	2.096 ± 0.149	-0.008 ± 0.069	0.907
	C12	1.016 ± 0.040	2.201 ± 0.089	-0.006 ± 0.039	0.969
	C15	1.014 ± 0.062	2.143 ± 0.134	-0.007 ± 0.061	0.926
	C23	1.026 ± 0.017	2.421 ± 0.046	-0.003 ± 0.017	0.995
Collagen IV	U-251 MG	0.571 ± 0.038	0.361 ± 0.200	0.000 ± 0.014	0.988
	C-	0.438 ± 0.024	0.049 ± 0.140	0.000 ± 0.010	0.991
	C12	0.509 ± 0.030	0.229 ± 0.167	0.000 ± 0.012	0.990
	C15	0.474 ± 0.086	0.125 ± 0.118	0.000 ± 0.009	0.994
	C23	0.604 ± 0.085	1.289 ± 0.597	0.000 ± 0.019	0.966
Vitronectin	U-251 MG	-	-	-	-
	C-	-	-	-	-
	C12	0.493 ± 0.059	0.071 ± 0.317	-	0.955
	C15	-	-	-	-
	C23	-	-	-	-

Ad_{max} and Ad_{min} refer to maximum and minimum O. D. values associated with adhesion for each condition. K_{Ad} is a constant that reflects the time in which half the adhesion response value was measured. The goodness of fit is expressed by the adjusted correlation coefficient (R^2_{adj}).

Supplementary Table 3: Non-linear regression to the Hill equation parameters regarding the TMZ susceptibility assay

Cell line	IC50 (mM)	$R^2_{adj.}$
U-251 MG	7.27 ± 0.50	0.913
C-	-	-*
C12	3.77 ± 0.29	0.792
C15	3.62 ± 0.15	0.910
C23	1.16 ± 0.02	0.834

*C- could not be adjusted to the Hill equation. Control GBM cell lines' (U-251 MG and C-) and GPC1 knocked-down GBM clones' (C12, C15, and C23) experimental values to the TMZ susceptibility assay were adjusted to the Hill equation to obtain IC50. The goodness of fit is expressed by the adjusted correlation coefficient (R^2_{adj}).

Supplementary Table 4: shRNA sequences

Gene	Source	Catalog number	shRNA sense sequence
GPC1	Dharmacon	V3SH7590-226429024	AGCTCTTCCTGCTGACCTT
GAPDH	Dharmacon	S07-001000-01	GTGTGAACCATGAGAAGTA
Non-target shRNA	Dharmacon	S07-005000-01	TGGTTTACATGTTGTGTGA

Supplementary Table 5: Primers used in RT-qPCR

Gene (human)	Forward (F) and reverse (R) primers (5′-NNN-3′)
Glypican-1	F: 5′-TATTGCCGAAATGTGCTCAAGGGC-3′<br>R: 5′-ATGACACTCTCCACACCCGATGTA-3′
Glypican-2	F: 5′-TCCTTTCTGGTTCACACACTGGCT-3′<br>R: 5′-ACAGGCCATTGAATATGAGGGCGT-3′
Glypican-3	F: 5′-TGAAAGTGGAGACTGCGGTGATGA-3′<br>R: 5′-TCCCGAGGTTGTGAAAGGTGCTTA-3′
Glypican-4	F: 5′-TCGGAGATGTCCCTCGCAAATTGA-3′<br>R: 5′-TCTTCAACAGGGCATGGGTACACT-3′
Glypican-5	F: 5′-ACTGGCATGCATATATCCGGTCGT-3′<br>R: 5′-TGAGGTGAGCCTGTAACACAGCAT-3′
Glypican-6	F: 5′-CTTCATTGCTGCCAGGACCTTTGT-3′<br>R: 5′-AGCCCTTCATGACGTTGAGACAGT-3′
Syndecan-1	F: 5′-AGGGCTCCTGCACTTACTTGCTTA-3′<br>R: 5′-ATGTGCAGTCATACACTCCAGGCA-3′
Syndecan-2	F: 5′-AACTTCTGCCGTAGCTCCCTTTCA-3′<br>R: 5′-AGGCTGCTCTCTGAAGCTCTTCTT-3′
Syndecan-3	F: 5′-AAGGAGGTGCTCGTAGCTGTGATT-3′<br>R: 5′-TCCTGCTTGTCAGGCTTCTGGTAT-3′
Syndecan-4	F: 5′-CCAGTTTGATGTTGCTGGGTGGTT-3′<br>R: 5′-AGCCCTAGAGCCTGAAGAAAGCAA-3′
Wnt-3a	F: 5′-CCATCCAGCTACAGGAGAGATA-3′<br>R: 5′-TGGACCTCTCTTCCTACCTTT-3′
Wnt-5a	F: 5′-CCCAGGACCCGCTTATTTATAG-3′<br>R: 5′-GGTTCCGGTTGCAATTCTTG-3′
Wnt-7a*	F: 5′-CTTCGGGAAGGAGCTCAAA-3′<br>R: 5′-GCAATGATGGCGTAGGTGA-3′
β-catenin	F: 5′-CTTCACCTGACAGATCCAAGTC-3′<br>R: 5′-CCTTCCATCCCTTCCTGTTTAG-3′
Metalloproteinase-2*	F: 5′-ATAACCTGGATGCCGTCGT-3′<br>R: 5′-AGGCACCCTTGAAGAAGTAGC-3′
Metalloproteinase-9*	F: 5′-GAACCAATCTCACCGACAGG-3′<br>R: 5′-GCCACCCGAGTGTAACCATA-3′
PPIB	F: 5′-GATGGCACAGGAGGAAAGAG-3′<br>R: 5′-AGCCAGGCTGTCTTGACTGT-3′
GAPDH	F: 5′-TCGACAGTCAGCCGCATCTTCTTT-3′<br>R: 5′-ACCAAATCCGTTGACTCCGACCTT-3′
β-actin	F: 5′-ACCAACTGGGACGACATGGAGAAA-3′<br>R: 5′-TAGCACAGCCTGGATAGCAACGTA-3′

*KAMINO, M. et al. Wnt-5a signaling is correlated with infiltrative activity in human glioma by inducing cellular migration and MMP-2. Cancer Sci, v. 102, n. 3, p. 540–8, Mar 2011.
